# Increased burrow oxygen levels trigger defensive burrow-sealing behavior by plateau zokors

**DOI:** 10.1038/s41598-021-98551-2

**Published:** 2021-09-27

**Authors:** Bin Chu, Yongliang Tian, Jianwei Zhou, Zhuangsheng Tang, Kechi Dong, Limin Hua

**Affiliations:** 1 College of Grassland Science, Gansu Agricultural University, Lanzhou, 730070 China; 2 Engineering and Technology Research Center for Alpine Rodent Pest Control, National Forestry and Grassland Administration, Lanzhou, 730070 China; 3 Key Laboratory of Grassland, Ministry of Education, Lanzhou, 730070 China; 4 Station of Forage and Feed Station, Dazhou, 635000 China

**Keywords:** Ecology, Zoology

## Abstract

Defensive behaviors are a response to immediate and potential threats in the environment, including abiotic and biotic threats. Subterranean rodents exhibit morphological and physiological adaptions for life underground, and they will seal with mounds and additional plugs when their burrow opened. However, little is known about the factors driving this defensive behavior. In this study, we selected a subterranean rodent, plateau zokor (*Myospalax fontanieri*), as a species to investigate (both in the laboratory and in the field) the possible factors responsible for burrow-sealing behavior. Our results showed that: (1) In the laboratory, the burrow-sealing frequency of plateau zokor in response to five factors were as follows: oxygen (52.63%) > light (34.58%) > temperature (20.24%) > gas flow (6.48%) > sound/control (0%). Except for light, the burrow-sealing frequency in response to other factors was significantly lower than that in response to oxygen (*P* < 0.05). (2) Burrow-sealing behavior in response to each treatment did not differ significantly between males and females in the laboratory experiment. (3) In the field, during the animal’s active periods in both the cold and warm season, the burrow-sealing frequency under the oxygen treatment was higher than that under the light and temperature treatments. Plateau zokors were found not to be sensitive to these treatments during their inactive periods during both the cold and warm season. (4) The latency to reseal the burrow showed no obvious differences between each treatment both in the laboratory and in the field. In conclusion, the main factor that influences the burrow-sealing behavior of plateau zokors is the variation in oxygen concentration, and this defensive behavior is related to their activity rhythm.

## Introduction

Defensive behaviors are a set of responses to threat stimuli and situations that have evolved on the basis of their adaptiveness in reducing harm to the threatened organism^1^. For the vast majority of species, these threat-response behaviors do not rely on specific experience, but instead are based on instinct. A defensive repertoire is constructed of such behaviors, each of which has proved successful in response to particular types of threats in particular situations^2^. Biotic threats, such as predators and conspecifics, may involve an intricate arms race that ultimately facilitates the development of elaborate patterns of species-specific defensive behaviors, whereas abiotic environmental threats, such as floods or fires, cause relatively few and simple defenses^1,3^.

Subterranean rodents are small mammals that live in underground tunnels and undergo most of their life activities below the soil surface^4^. Across the globe, subterranean rodents, with at least 140 species belonging to 20 genera of 8 families, occur in all continents except Australia and Antarctica^5^. Compared to aboveground rodents, subterranean habitats are considered more stable and buffered than aboveground habitats. This shelter not only protects these animals from predators, but also from environmental fluctuations or extremes taking place above the ground^6^. However, sometimes, their tunnels are opened inadvertently because of trampling by livestock or researchers using open-hole assessments to monitor their population dynamics^7,8^. These opened tunnels, which constitute a threat to the occupants, will be sealed by the animals with additional plugs. Although most researchers have observed this defensive behavior in subterranean rodents, little is known about the factors driving this defensive behavior.

Previous studies have focused on the effect of wind and light on burrow-sealing behavior. Yu and Qian^9^ concluded that wind stimulated the occurrence of such behavior in Gansu kozor (*Myospalax epsilanus Thomas*). Han^10^ suggested that wind and light have a large influence on the burrow-sealing behavior in Transbaikal zokor (*Myospalax psilurus*). However, Chai et al.^11^ found that this behavior in Transbaikal zokor was unrelated to wind and light. In general, burrow systems are characterized by the absence of light, relatively invariant temperatures, low ratios of oxygen to carbon dioxide, and gas flow is generally reduced relative to the surface habitat^12^. In facing these challenges, the morphology of subterranean rodents has been molded by convergent evolution to the same extent as their physiology. For instance, the dark environment has resulted in small eye sizes that limit the image size on the retina, resulting in poor image quality and visual acuity, but they can still detect ambient light levels for photoperiodism^13^. In subterranean ecotypes, olfaction and audition play an important role in the lives of these animals that have successfully adapted to life underground. The role of olfaction is one not only of finding food, but also in recognizing familiar individuals and choosing mates^14,15^. However, the spread of predator odor over long distances in the subterranean environment is restricted^14^ and, besides, predators are generally unable to open tunnels to prey upon these species^16,17^. Compared to epigeic generalized rodents, the hearing range of subterranean rodents is not restricted, rather, it is shifted towards lower frequencies^18^. More importantly, poor ventilation in subterranean burrows leads to poor levels of oxygen and an abundance of carbon dioxide, and, as such, these species have evolved physiological strategies enabling their respiratory and cardiovascular systems to cope with hypoxia and hypercapnia^19,20^. For instance, they have a large oxygen carrying capacity facilitated by elevated hemoglobin concentrations, a high intrinsic affinity for oxygen, and large concentration of red blood cells^21^. Moreover, the critical O_2_ (pO_2_) partial pressure levels in these mammals are low and metabolic rates can be maintained at low pO_2_^22^. Therefore, when a burrow is opened, the atmosphere inside the burrow will become richer in oxygen, which may result in harmful effects on these species. Clearly, all of these factors (oxygen, light, temperature, sound, gas flow) need to be considered as possible determinants of burrow-sealing behavior, both in the laboratory and in field experiments.

In the present study, we selected plateau zokors, belonging to the genus *Myospalax*^5^, as our species of subterranean rodent to study. Most plateau zokors live in alpine meadows on the eastern Qinghai–Tibetan Plateau^23^, and like other subterranean rodents, they possess morphological, physiological, and behavioral adaptions for digging and living in the underground environment^24^. Plateau zokors construct complicated burrow systems that consist of one or two main nests, foraging and transportation tunnels, food store caches, and blind endings. When constructing tunnels, the animals move the loosened soil to the surface and deposit it in mounds^23^. In this study, we conducted laboratory experiments to determine the effects of oxygen, light, temperature, gas flow and sound on the burrow-sealing behavior of plateau zokors. In addition, field experiments were conducted during both the cold and warm season of 2016. The aims of this research were to (1) reveal the determinants of burrow-sealing behavior in plateau zokor, (2) determine whether the latency to reseal the burrow is the difference between the responses to different treatments, (3) determine whether burrow-sealing behavior differs between the sexes, and (4) determine whether activity rhythm has an influence on burrow-sealing behavior in the field.

## Materials and methods

All experimental procedures were permitted by the Institutional Animal Care and Usage Committees of the Grassland Science College of Gansu Agricultural University (GSC-IACUC-2015-0011). Our experiments were conducted according to their guidelines, which are in accordance with the Guide for the Care and Use of Laboratory Animals (the Constitution of Experimental Animal Ethics Committee of Gansu Agricultural University). All experiments were performed in accordance with ARRIVE guidelines.

### Animals and laboratory conditions

Adults of both sexes (three males and three females) were captured in April 2015. Specifically, the animals were captured in Mayin Tan (37°12′N, 102°46′E; Tianzhu Tibetan Autonomous County, China) using live traps^25^ set at fresh surface mounds. The individuals were then transported to the laboratory and housed in an acrylic box with a pipeline covered with soil. The box and pipeline were covered by black cloth to simulate the dark environment of plateau zokors. The temperature in the room was maintained between 20 and 25 °C. Food was supplied daily and consisted of potatoes, lettuce and carrot. After three days of acclimatization to the laboratory the animals were used in the different experiments. Our laboratory is located 2 km away from the field site. At the end of the experiments all animals were returned to the capture site in good health.

### Laboratory testing arena

**Figure 1:**
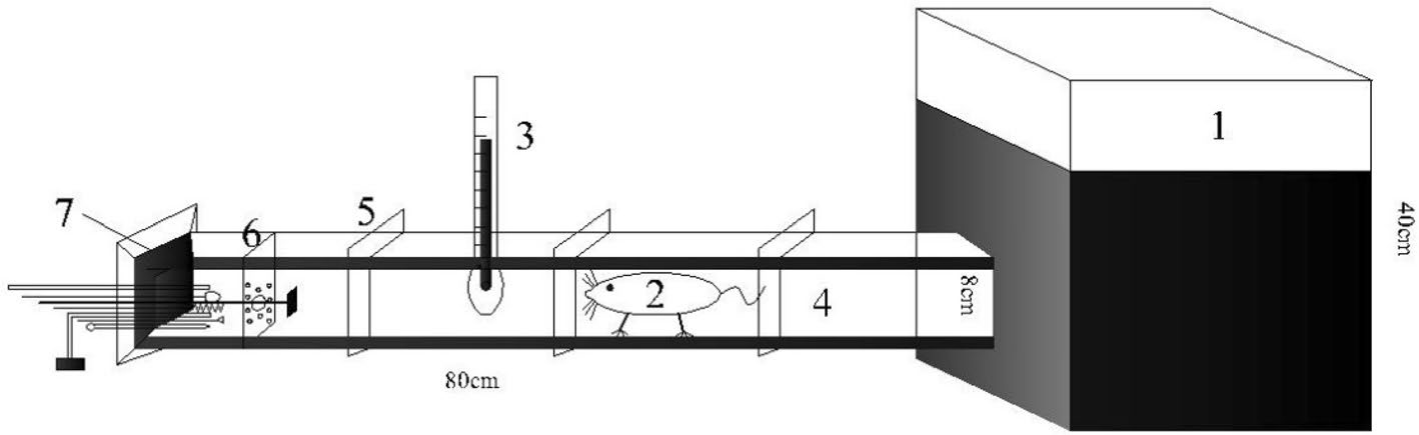
Schematic drawing of the setup used to test burrow-sealing behavior in plateau zokors in the laboratory. (1) acrylic box covered with soil 30 cm in depth; (2) experimental animal; (3) mercury thermometer; (4) transparent Perspex tube; (5) the pipe’s support clip; (6) wire mesh (8 cm × 8 cm × 0.5 cm); (7) rubber stopper (8 cm × 8 cm × 5 cm).

The experimental setup for the laboratory experiments was as follows (Fig. 1): A transparent Perspex tube (8 cm × 8 cm × 80 cm) was joined to the side of the dark acrylic box (40 cm × 40 cm × 40 cm). A rubber stopper was inserted into one end of the tube to avoid effects from the external environment. Treatment apparatus was placed into the rubber stopper (see “Laboratory treatment apparatus” section, below), and, to avoid the apparatus being damaged by the animals, wire mesh (8 cm × 8 cm × 0.5 cm) was placed about 15 cm from one end of the tube. A mercury thermometer was inserted into the tube in the middle to monitor the tube’s temperature. Between experiments with different animals, the box and tube were wiped with 95% alcohol and then with distilled water.

### Laboratory treatment apparatus

**Figure 2:**
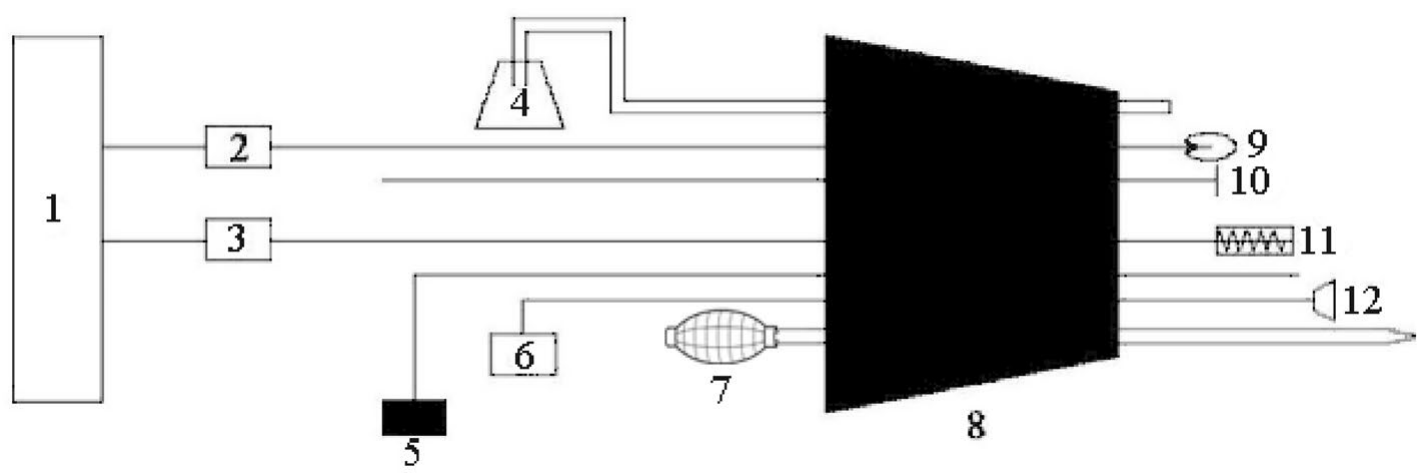
Schematic diagram of the rubber stopper used to simulate the entrance plug of the burrow. (1) power supply; (2) light bulb switch; (3) electric wire switch; (4) oxygen cylinder; (5) in situ three-parameter soil gas analyzer; (6) voice recorder; (7) negative pressure drainage device; (8) rubber plug; (9) LED bulb; (10) the iron rod; (11) heating cord; (12) AVOXIVY speaker with 5 cm diameter.

A rubber stopper with seven holes was used for plugging one end of the tube (Fig. 2). The oxygen concentration, light, temperature, sound and gas flow were considered in this design.

#### Oxygen treatment

To avoid the oxygen that was delivered into the tube causing the gas to flow too strongly, become drier, and create a sound, a steel oxygen cylinder and thin hose (0.3 cm in diameter) were selected, and one end of the hose was connected directly to the oxygen cylinder with a humidifier bottle, while the opposite end was inserted into the rubber stopper (Fig. 2). Before beginning the experiment, we allowed the oxygen cylinder to sit for two hours at laboratory temperature to remove any temperature effects. A three-parameter soil gas analyzer (13.05.03Pro, Shanghai SAFE Biotech Co., Ltd, China) was used to monitor the oxygen concentration in the tube (Fig. 2).

#### Light treatment

The average light intensity—that is, 360 Lux from 8:00 am to 8:00 pm—was measured in the field. One end of a wire was connected to an LED light (1 Watt), and the other end to the power supply (Fig. 2).

#### Temperature treatment

The temperature in the burrow entrance in the field was about 3 °C warmer than that at a tunnel depth of 10 cm. As such, one end of a wire was connected to a heater strip and the other end to the electrical power supply (Fig. 2). A thermometer was inserted into the tube to monitor the temperature inside the tube (Fig.1). During the experiment period in the laboratory, we switched on or off to make sure the relatively constant temperature inside the tube. The temperature range inside the tube was 3.2 ± 0.27 °C .

#### Sound treatment

When a burrow is opened, wind whistle can be produced around the burrow entrance. Accordingly, a voice recorder (PCM-D50, frequency response 50 Hz–40 kHz, Sony, Japan) was placed at the burrow entrance in the field to record the burrow-entrance sound, the duration of which was 30 min. In the laboratory, the two ends of a wire were connected to an AVOXIVY loudspeaker (diameter: 5 cm; impedance: 4 Ω; 50 Hz–20 kHz) and a voice recorder, respectively (Fig. 2). The recorded sound was played back with a 60 dB sound pressure level, as measured at the burrow entrance in the field (XL2 sound level meter, Nti Audion, Switzerland). The sound was repeatedly played within one hour.

#### Gas flow treatment

To avoid ambient atmosphere entering the tube, a negative pressure drainage ball with plastic tube (12 cm long, 2 cm in diameter) connected the tube through a rubber stopper (Fig. 2). The tunnel gas was inhaled by the ball, then we pinched the ball to blow the gas into the tunnel as gas flow treatment.

### Field treatment apparatus

**Figure 3:**
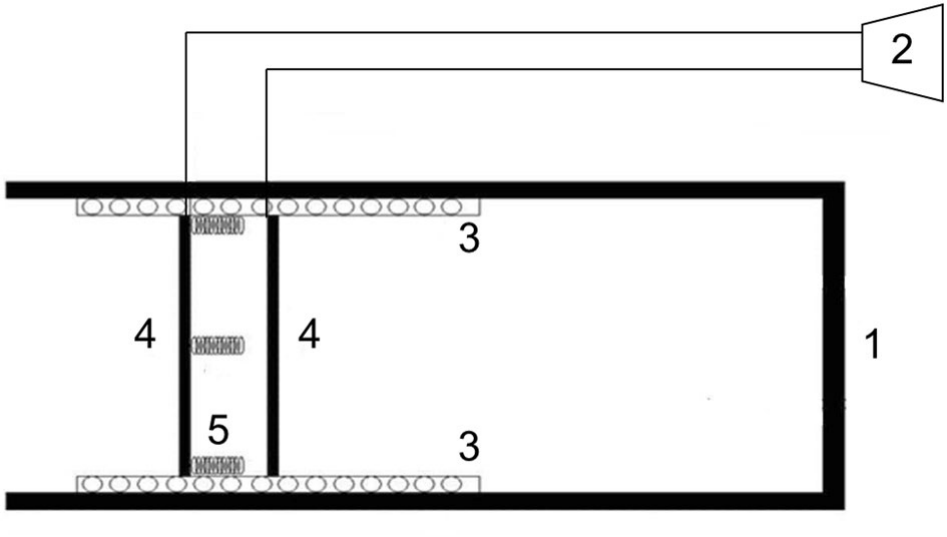
Schematic drawing of the apparatus used to test the burrow-sealing behavior of plateau zokor in the field. (1) tube; (2) loudspeaker; (3) slide rail; (4) metal plate; (5) coiled metal springs.

**Figure 4:**
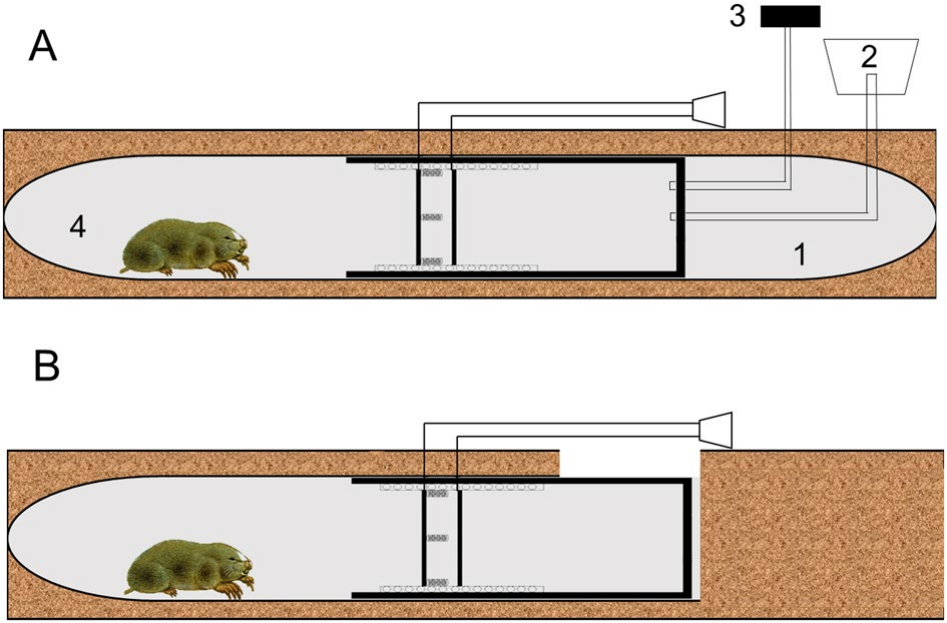
(**A**) Schematic drawing of the apparatus used in the oxygen treatment placed in the tunnel of the plateau zokor. (**B**) Schematic drawing of the apparatus used for the temperature and light treatments placed in the tunnel of the plateau zokor. (1) tunnel of the plateau zokor; (2) oxygen cylinder; (3) three-parameter soil gas analyzer; (4) plateau zokor.

For the field experiment, the apparatus consisted of a tube (40 cm long, 8 cm in diameter) and an alarm device. The alarm device was made up of a loudspeaker, two slide rails (15 cm long), two metal plates (approximately 7 cm in length and 3 cm in width), and three coiled metal springs (5 cm long, 2 cm in diameter). The three springs were joined to one of the metal plates, while the other metal plate was fixed on the slide rails. The two metal plates were touched by the plateau zokor when it was plugging, which triggered the alarm device, thus enabling us to know whether or not burrow-sealing behavior was occurring (Fig. 3). The aluminum tube with an oxygen device was embedded into the burrow. The soil covering the tube served as an excellent insulator, buffering the tube from the aboveground temperature (Fig. 4A). A steel oxygen cylinder and thin hose (0.3 cm in diameter) were applied by connecting one end of the hose directly to the oxygen cylinder with a humidifier bottle, and then the opposite end of the hose was inserted into the tube (Fig. 4A). A three-parameter soil gas analyzer (13.05.03Pro, Shanghai SAFE Biotech Co., Ltd, China) was used to monitor the oxygen concentration in the tube (Fig. 4A). Allowing sunlight to enter the burrow, a glass bottle, open at one end but closed at the other, was embedded into the burrow. We also used soil to cover the bottle, and there was a 5 cm gap at the surface (Fig. 4B). The aluminum tube with high thermal conductivity was embedded into the burrow. Again, we used soil to cover the bottle and retained a 20 cm gap (Fig. 4B).

### Procedure

In the laboratory experiment, we tested three males and three females for their responses to each treatment. To avoid generating stress and habituation to treatments, zokors were tested for 12 h each day and there was one hour interval between treatments, and five days interval between round of testing for the same individual (Table 1). We performed a control experiment in which a rod was inserted into the burrow but no further treatment was applied, which allowed us to evaluate whether it was the treatment that was causing the burrow-sealing behavior. Before beginning treatment experiment, each zokor was tested 24 times (12 h × 2 days) under the control experiment. We determined the rod movement as occurrence of burrow-sealing behavior.

**Table 1:** Times of the experiments for each treatment in the laboratory simulation.

Testing time	Round of testing				
	1st	2nd	3rd	4th	5th
08:00–09:00	O	T	S	L	A
10:00–11:00	T	O	L	A	S
13:00–14:00	L	S	A	O	T
16:00–17:00	S	A	O	T	L
19:00–20:00	A	L	T	S	O

This table only represents the times of experiments for one individual from the six individuals that were used in the experiments. O, T, L, S and A represent the oxygen, temperature, light, sound and gas flow treatments, respectively.

In the field experiment, we tested three zokors (one male, two females), and six zokors were caught in the cold season and warm season (three males and three females, respectively). We then fastened radio collars (Ag357, Biotrack, Ltd., UK) to each captured individual to allow us to track the position in foraging tunnels of each zokor. Each zokor was used three times in the experiments under each treatment, and, after finishing each experiment, we changed the position of the foraging tunnel to ensure the test tunnel was not an abandoned tunnel. According to radio-tracking data, the straight-line distance between the test tunnel and the nest for each treatment was about 5 m. We conducted a control experiment that whether plateau zokor move to the test tunnel or not during the time between treatments. In the cold season, from 4 October 2015 to 2 November 2015, the burrow-sealing behavior of each zokor was tested under different treatments during their active time (12:00–18:00) and inactive time (09:00–11:00) for a total of 27 days (Table 2). The same was done in the warm season but for a total of 18 days from 15 May 2016 to 5 June 2016, in which the active time was 14:00–20:00 and the inactive time was 08:00–13:00 (Table 2).

**Table 2:** Times of the experiments for each treatment in the active and inactive periods of plateau zokors during the warm and cold season.

Season	Activity pattern	Testing time	Testing day								
			1st	2nd	3rd	4th	5th	6th	7th	8th	9th
Warm season	Inactive time	08:00–09:30	O	T	L						
		10:00–11:30	T	L	O						
		12:00–13:30	L	O	T						
	Active time	14:00–15:30	O	T	L						
		16:00–17:30	T	L	O						
		18:00–19:30	L	O	T						
Cold season	Inactive time	09:00–11:30	O	T	L	O	T	L	O	T	L
	Active time	12:00–13:30	O	T	L						
		14:00–15:30	T	L	O						
		16:00–17:30	L	O	T						

This table only represents the times of experiments for one individual from the six and three individuals used in these experiments in the warm and cold season, respectively. O, T and L represent the oxygen, temperature, and light treatments, respectively.

### Data analysis

The occurrence of burrow-sealing was recorded as “1”, and non-sealing was recorded as “0”. The frequency of burrow-sealing was the number of times the burrow was sealed divided by the total number of experiments for each treatment^26^, and we considered the frequency for each individual as a replicate. The latency to reseal the burrow was the period from the start of the treatment to the sealing of the burrow, and we considered each instance of latency to reseal the burrow as a repeat. The latency to reseal the burrow for non-sealing under each treatment was unavailable data and was therefore removed. The presence of a normal distribution in the initial data was determined using the Kolmogorov–Smirnov test. All data followed a normal distribution. A comparison of males and females in their frequency of sealing the burrow and in their latency to reseal the burrow under each treatment was performed with an independent-samples T-test. Multiple comparisons were made for the frequency of burrow-sealing and the latency to reseal the burrow under different treatments by using the least significant difference method at the significance level of *P* = 0.05. In the field experiment, the number of replicates was fewer than three for frequency and the latency to reseal the burrow, we did not conduct multiple comparisons.

Preliminary statistical analysis of the data was performed using Excel 2013 and SPSS 19.0. All the figures and tables were produced in GraphPad Prism 8.0 and Excel 2013.

## Results

### Responses of burrow-sealing frequency and the latency to reseal the burrow to different treatments in the laboratory experiments

**Figure 5:**
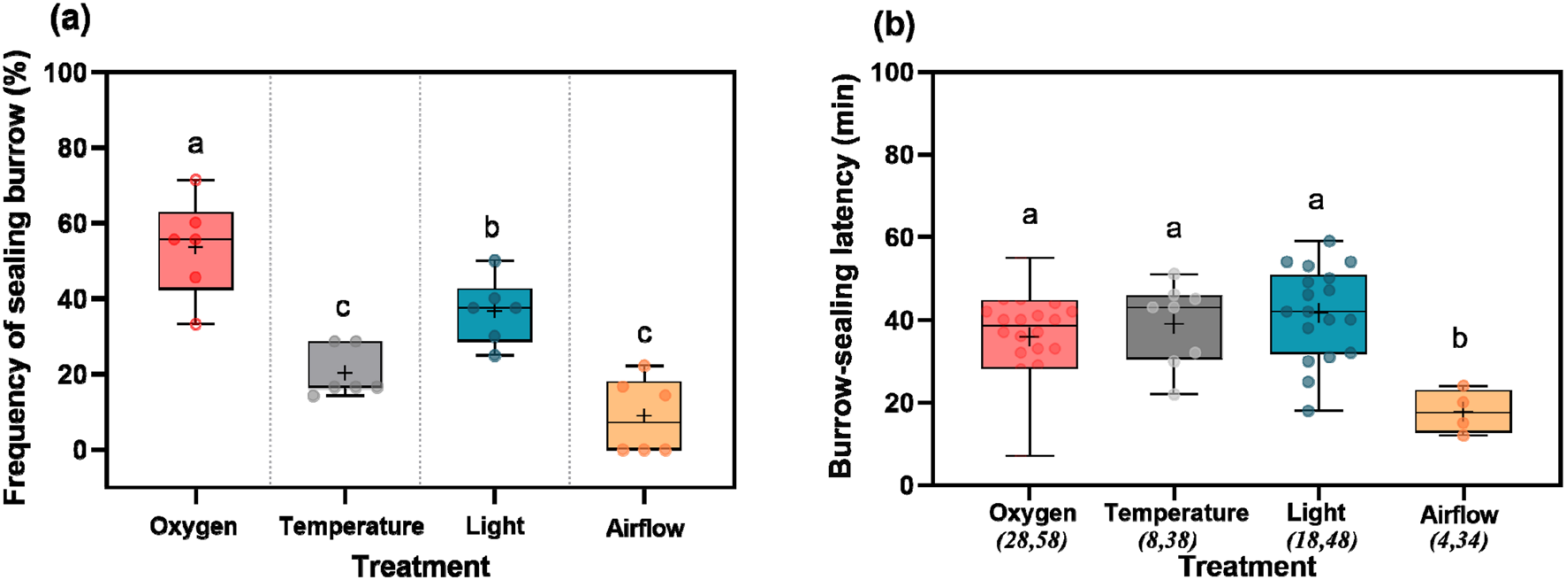
The frequency of burrow-sealing and the latency to reseal the burrow in response to different treatments. The upper and lower horizontal lines of the boxes represent the maximum and minimum values, respectively. The black plus signs and the central horizontal lines represent the mean and median, respectively. Different colored dots in (**a**) represent the number of zokor individuals (N = 6), while in (**b**) they represent the number of burrow-sealing events. The left-hand values of the pairs of italic numbers in brackets on the horizontal axis in (**b**) denote the number of burrow-sealing events, while the right-hand values are the number of experiments. The results of the statistical tests are displayed in the text. Different lowercase letters indicate a significant difference (*P* < 0.05), while the same lowercase letter indicates a non-significant result.

Under the sound and control treatment, no burrow-sealing events were observed in 30 tests. Comparing the different treatment groups, the frequency of burrow-sealing was highest in the oxygen treatment (52.63%), and significantly higher than in the other treatments (Fig. 5a). The lowest frequency of burrow-sealing (6.48%) was in the gas flow treatment. We observed 28 events of burrow-sealing in the oxygen treatment during 58 experiments and the average latency to reseal the burrow was 35.85 min, which was not significantly different to the temperature and light treatments (Fig. 5b). Although the latency to reseal the burrow in the gas flow treatment was significantly lower (17.75 min) than in the other treatments, only four events of burrow-sealing in 34 trials were observed (Fig. 5b).

### Responses of burrow-sealing frequency and latency to reseal the burrow in males and females to different treatments in the laboratory

**Figure 6:**
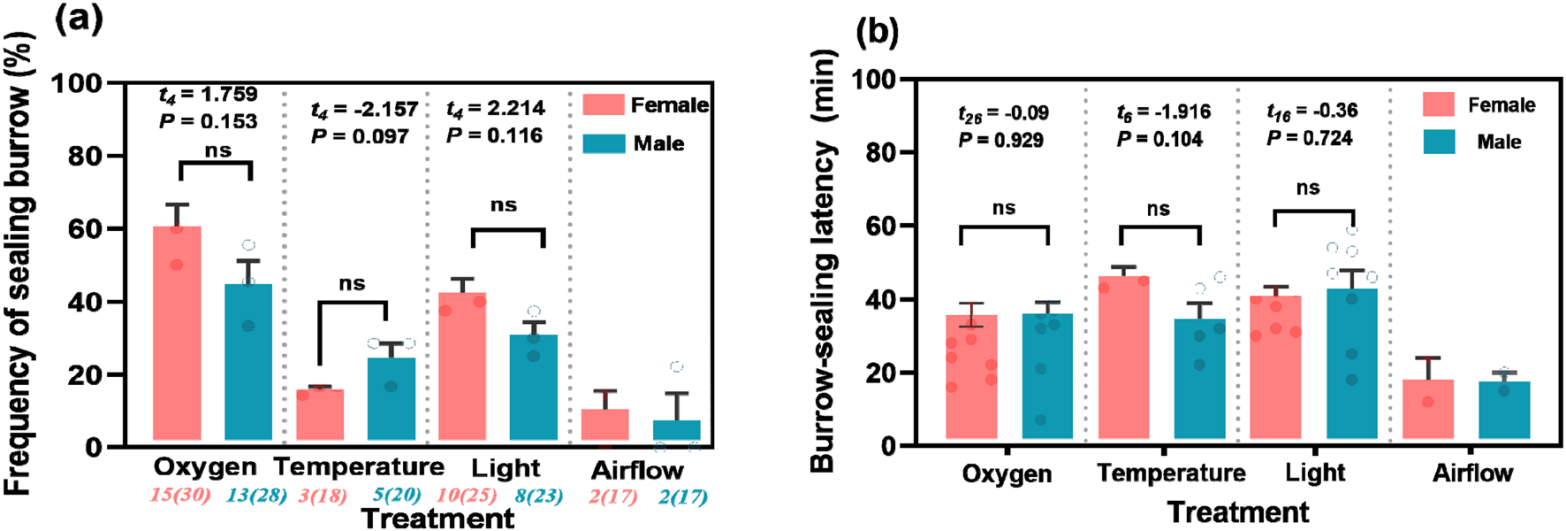
The frequency of burrow-sealing and the latency to reseal the burrow in response to different treatments between the sexes. The results of independent-samples T-tests for the differences (*P* < 0.05) between females (N = 3) and males (N = 3) are displayed. Pink bars and blue bars represent females and males, respectively; and pink and blue dots in (**b**) represent the number of occurrences of sealing burrows in females and males, respectively. The italic numbers on the horizontal axis in (**a**) refer to the number of burrow-sealing events (numbers not in brackets) and the number of experiments (numbers in brackets), and “ns” in both panels denotes “non-significant”.

Burrow-sealing behavior in male and female zokors was not observed in the sound and the control. The frequency of burrow-sealing and the latency to reseal the burrow showed no differences between the sexes under the different treatments (Fig. 6). Specifically, the frequency of burrow-sealing in response to oxygen for females and males was 60.00% and 44.78%, respectively, and was higher than that in response to other factors. Compared with other factors, although the latency to reseal the burrow in response to gas flow was lowest in both females (18.00min) and males (17.50 min), the frequency of burrow-sealing was only two times in males and females, respectively. However, the latency to reseal the burrow in response to oxygen did not show the same trend.

### Response of frequency and latency of burrow-sealing to different factors in the field

In the laboratory experiments we found that plateau zokors were sensitive to oxygen, temperature and light, and there were no differences between the sexes in their response to each treatment. Therefore, we considered these factors as treatments for the field experiment and did not consider the difference between the sexes. Plateau zokors did not move to the test tunnel during the time between treatments in the field. Regardless of whether in the cold or warm season, the number of burrow-sealing events in the inactive period was lower than that in the active period (Table 3). In the warm season, the frequency of burrow-sealing in response to oxygen was significantly higher than that in response to temperature during the active period (*df* = 2, *P* = 0.01, Table 3), and we found no significant difference between oxygen and light (*df* = 2, *P* = 0.05, Table 3). In the inactive period during the warm season, the frequency of burrow-sealing was low for each treatment (Table 3). In the cold season, the frequency of burrow-sealing in response to oxygen was highest in the active period, and the frequency of burrow-sealing in response to temperature and light was 0 and 1 in the active period, respectively. Therefore, we did not conduct any statistical analysis. In the inactive period during the cold season, we did not find any burrow-sealing behavior under any of the treatments.

**Table 3:** Burrow-sealing frequency in the field under different treatments.

Season	Activity pattern	Frequency of burrow-sealing (%)			*F*	*P*
		Oxygen	Temperature	Light		
Warm season	Active time	61.11 ± 5.55a	27.78 ± 10.24b	38.88 ± 5.55ab	5.18	0.01
		11 (18)	5 (18)	7 (18)		
	Inactive time	11.11 ± 7.02	11.11 ± 7.02	5.55 ± 5.55	–	–
		*2 (18)*	2 (18)	1 (18)		
Cold season	Active time	33.32 ± 19.24	0	11.11 ± 11.10	–	–
		4 (9)	0 (9)	1 (9)		
	Inactive time	0	0 0	0	–	–
		0 (9)	(9)	0 (9)		

Italic numbers not in brackets denote the number of burrow-sealing events, while those in brackets are the number of experiments. Different lowercase letters indicate a significant difference (*P* < 0.05), while the same lowercase letter indicates a non-significant result.

The latency to reseal the burrow in response to the oxygen, temperature and light treatments in the inactive period during the cold season was absent because no burrow-sealing was observed. Under the oxygen treatment, the latency to reseal the burrow was shortest in the active period during the cold and warm season (Table 4). In the warm season, the latency to reseal the burrow in response to the light treatment was shorter than in the other treatments (Table 4).

**Table 4:** Burrow-sealing latency in the field under different treatments.

Season	Activity pattern	Time to seal burrow (min)			*F*	*P*
		Oxygen	Temperature	Light		
Warm season	Active time	61.08 ± 3.91	74.25 ± 0.62	62.67 ± 3.79	3.38	0.065
	Inactive time	66.00	87.00	54.00	–	–
Cold season	Active time	35.50 ± 1.89	–	54.00	–	–
	Inactive time	–	–	–	–	–

## Discussion

We examined the burrow-sealing behavior of plateau zokors in response to oxygen, light, temperature, sound and gas flow, both in the laboratory and in field experiments. The results indicated that burrow-sealing behavior in response to each treatment did not differ significantly between males and females. We also found that oxygen, light and temperature had an effect on burrow-sealing in the laboratory experiment—especially a change in the oxygen concentration. Accordingly, we selected these three factors to verify the results in the field and found that oxygen and light influenced the frequency of burrow-sealing in the active period during the cold and warm seasons, but that plateau zokors were not sensitive to these treatments in the inactive period, again during both seasons. Therefore, based on laboratory and field experiments, we are able to conclude that the oxygen concentration has a major effect on the burrow-sealing behavior of plateau zokors, and that there are no differences in this regard between males and females. The latency to reseal the burrow showed no obvious differences between each treatment. We also suggest that this defensive behavior is related to the activity rhythm of plateau zokors. Below, we discuss these findings in the context of the wider literature.

Both in the laboratory and in the field, oxygen had the main effect on the frequency of burrow-sealing in plateau zokors. About 11 million years ago, plateau zokors evolved physiological strategies enabling their respiratory and cardiovascular systems to cope with hypoxia^27,28^. Therefore, when the burrow atmosphere becomes richer in oxygen when the burrow is opened, harmful effects on these animals might arise. Indeed, an increased oxygen concentration in the ambient atmosphere is poisonous to all mammals, regardless of the baseline concentration that they are used to, bringing about symptoms such as drowsiness, anorexia, loss of weight, increasing dyspnea and cyanosis^29^. Furthermore, too much oxygen is toxic to the mammalian central nervous system owing to the excessive production and accumulation of reactive oxygen species^30^. Therefore, we infer that oxygen concentration within zokor’s tissues must be tightly regulated to avoid possible harmful effects, although the response to hyperoxia has not been studied in plateau zokors. However, burrow-sealing behavior in response to oxygen is dependent on the activity rhythm of plateau zokors. In the field, the burrow-sealing frequency in response to oxygen was very low in the inactive period during both the cold and warm season (Table 3). Because plateau zokors spend much of their time in their main nest (about 1.5–2.5 m underground) during their inactive period and possess a “deep sleep” phenomenon in their nest^31,32^, they are therefore not sensitive to oxygen during these periods.

In our study, we also found that light had a slight effect on burrow-sealing behavior after eliminating oxygen, temperature and other factors. Han^10^ also suggested that light might trigger burrow-sealing behavior in Transbaikal zokor. Subterranean rodents live in a dark environment for a long time and their eyes degenerate as a consequence^13^. Nevertheless, although they cannot see images, these rodents are able to detect photoperiods and differentiate light from dark^13^. We infer that plateau zokors may be able to perceive light in a dark tunnel during the process of tunnel inspection and seal the burrow entrance in response. Our results also indicated that sound from the burrow entrance does not induce plateau zokors to seal their burrow. In this regard, it is possible that the hearing of plateau zokors was not sensitive to the frequency of the sound recording. Indeed, compared to aboveground generalist rodents, their hearing range is not restricted but they are more sensitive to the low-frequency range^18^. Zhou ^33^ found that within the auditory range that evoked a response, from 250 to 4000 Hz, the average lowest sound that plateau zokor could hear was 53.9 ± 2.8 dB SPL (range: 41–71 dB SPL), although the sensitivity of plateau zokor to low-frequency sounds was the worst of the subterranean rodents that have been studied. Interestingly, in the laboratory, our results from the gas flow treatment yielded both the shortest latency to reseal the burrow and the lowest frequency of burrow-sealing events, which seems contradictory. However, we thought that a stress reaction could be the trigger of rapid burrow-sealing behavior in plateau zokors. The shorter distance (about 1 m) between the end of tube and the zokor’s nest probably made the zokor feel uncomfortable because of the gas flow. Therefore, they would act to seal their burrow rapidly. However, the lowest frequency of burrow-sealing in this instance may be beneficial to plateau zokors in terms of conserving energy. Because gas flow did not produce a negative physiological response owing to the negative pressure drainage ball with plastic tubing used in the laboratory.

In our study, we found that the frequency of burrow-sealing and the latency to reseal the burrow under different treatments did not differ between males and females in the laboratory experiments. In other words, both males and females will seal the burrow when it is opened. Unlike social subterranean rodents, plateau zokors are solitary animals^24^ and have their own home ranges. Thus, both males and females must rely on themselves to ensure all its own burrow systems are sealed. Furthermore, some researchers have found that both males and females have been captured when capturing zokors by using the opening–sealing burrow method^34^. Therefore, we confirm that opening the burrow entrance has the same influence on both males and females. However, we do not know whether different ages of individuals have similar responses to these treatments, which needs to be studied in future research.
